# Mesenteric origin hyalinized vascular pseudotumor mimicking duplicate acute appendicitis

**DOI:** 10.23938/ASSN.1135

**Published:** 2025-10-23

**Authors:** Hüseyin Taş, Furkan Karahan, Arzu Avcı

**Affiliations:** 1 Department of General Surgery Atatürk Education and Research Hospital Izmir Turkey; 2 Izmir Kâtip Çelebi University Izmir Turkey; 3 Department of General Surgery Erciş State Hospital Van Turkey; 4 Department of Pathology Atatürk Education and Research Hospital Izmir Turkey

**Keywords:** Appendicitis, Appendiceal, Duplication, Pseudotumor, Vascular, Hyalinized, Apendicitis, Duplicación del apéndice, Pseudotumor, Vascular, Hialinizado

## Abstract

Appendiceal duplication is an exceptionally rare anomaly, with an incidence ranging from 0.004% to 0.009%. Most cases are asymptomatic and remain undetected on imaging, with diagnosis usually established incidentally during laparotomy performed for other intra-abdominal conditions. The differential diagnosis includes cecal diverticulum, mesenteric adenitis, epiploic appendicitis, and neoplasms of the colon or appendix.

We report the case of a 22-year-old man whose preoperative findings and intraoperative appearance suggested appendiceal duplication. Histopathological examination, however, demonstrated acute appendicitis in one specimen and hyalinized vascular pseudotumor of mesenteric origin in the other. To the best of our knowledge, no prior reports describe this combination.

This case highlights the diagnostic challenge posed by unusual mesenteric lesions that can mimic rare congenital anomalies such as appendiceal duplication. Awareness of such entities may prevent misinterpretation and contribute to a more accurate understanding of appendiceal pathology.

## RESUMEN

La duplicación apendicular es una anomalía extremadamente rara con una incidencia que oscila entre el 0,004% y el 0,009%. A menudo asintomática, es difícil detectarla mediante técnicas de imagen y suele diagnosticarse durante laparotomías realizadas por otras patologías intraabdominales. El diagnóstico diferencial incluye divertículo cecal, adenitis mesentérica, apendicitis epiploica, y neoplasias malignas de colon y apéndice.

Los hallazgos clínicos pre- e intraoperatorios en el varón de 22 años aquí presentado hicieron sospechar la presencia de un apéndice duplicado. Sin embargo, el examen histopatológico reveló un apéndice con apendicitis aguda y un pseudotumor vascular hialinizado de origen mesentérico. No hemos encontrado una presentación similar en la literatura.

Este caso destaca el reto diagnóstico que suponen las lesiones mesentéricas que pueden imitar anomalías congénitas inusuales, como la duplicación apendicular. El conocimiento de estas entidades puede evitar malinterpretar los hallazgos, además de contribuir a un mayor conocimiento de la patología apendicular.

## INTRODUCTION

Appendiceal duplication is a rare congenital anomaly, with an estimated incidence of 0.004%-0.009% and approximately 140 reported cases to date^1^^,^^2^. Among these, presentations with acute appendicitis are particularly uncommon, with fewer than 15 cases described in the literature^3^. Preoperative diagnosis is difficult, as duplication is usually identified incidentally - either during imaging for unrelated abdominal conditions or more often intraoperatively during laparotomy.

Various classification methods are utilized for duplicated appendices, with the Cave-Wallbridge classification being the most widely accepted. This system categorizes duplication into four types according to the anatomical location of the appendix. Certain subtypes are also associated with other congenital anomalies, including colonic atresia, bladder malformations, and vertebral defects^4^^,^^5^. The clinical manifestations of duplicated appendicitis can resemble those of other abdominal pathologies. Reported cases have been misdiagnosed as appendiceal malignancy, colon adenocarcinoma, small bowel obstruction, and cecal diverticulitis^2^^,^^6^^,^^7^.

Here, we describe a case of mesenteric-origin hyalinized vascular pseudotumor in a young patient, initially suspected to represent duplicated appendicitis.

## CASE REPORT

**Figure 1:**
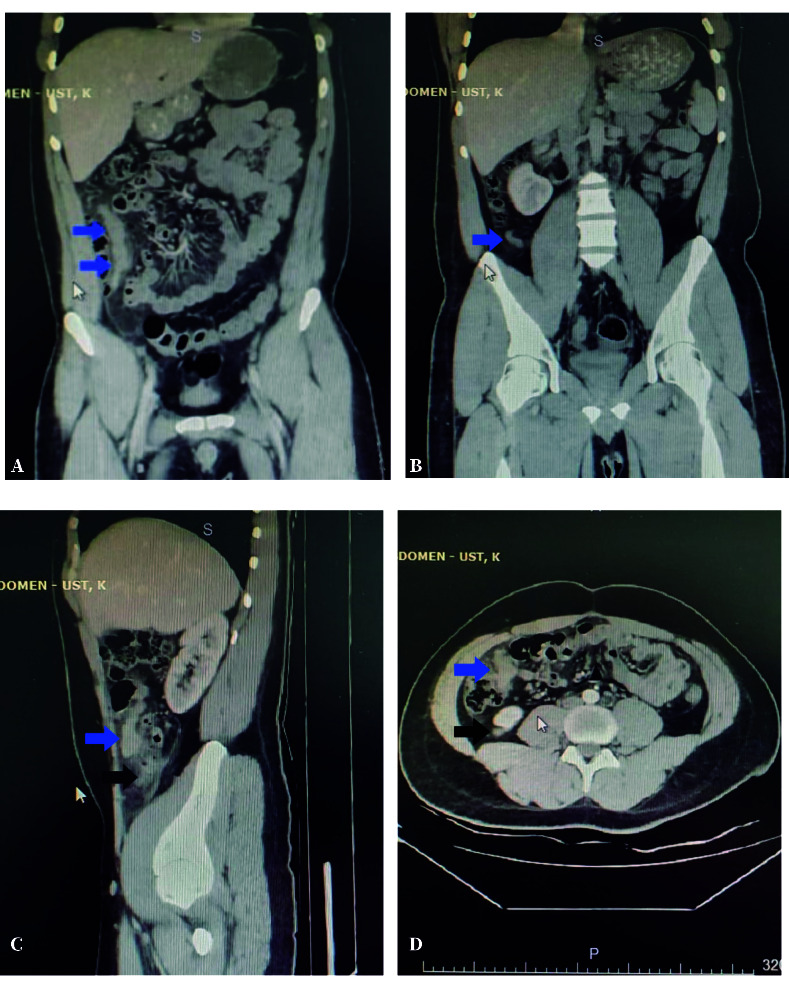
Contrast-enhanced computerized tomography images. **A.** Coronary view: blue arrows indicate a structure presumed to be an anteriorly located appendix. **B.** Coronary view: blue arrow indicates a retrocecal appendage. **C.** Sagittal and **D.** Axial views: the blue arrow indicates the anteriorly located structure, while the black arrow highlights the retrocecal appendage.

A 22-year-old male presented with abdominal pain persisting for 36 hours. Associated symptoms included nausea, anorexia, and fever. He had no known comorbidities and no history of abdominal surgery. On physical examination, localized tenderness was elicited at McBurney’s point, accompanied by peritoneal signs guarding and rebound tenderness. Urinalysis was unremarkable. Laboratory investigations revealed leukocytosis with a white blood cell count of 12.23 x 10^9^/L (reference range: 4.0-10.0), and elevated C-reactive protein at 82.8 mg/L (reference range: 0-5). Plain chest and abdominal radiographs were normal. Contrast- enhanced computed tomography (CT) demonstrated an 8 mm inflamed appendix extending retrocecally from the cecal apex, as well as an additional 18 mm inflamed tubular soft tissue density arising from the anterior cecum (Fig. 1). The terminal ileum appeared normal, and multiple reactive lymph nodes were observed in the ileocecal mesentery.

**Figure 2:**
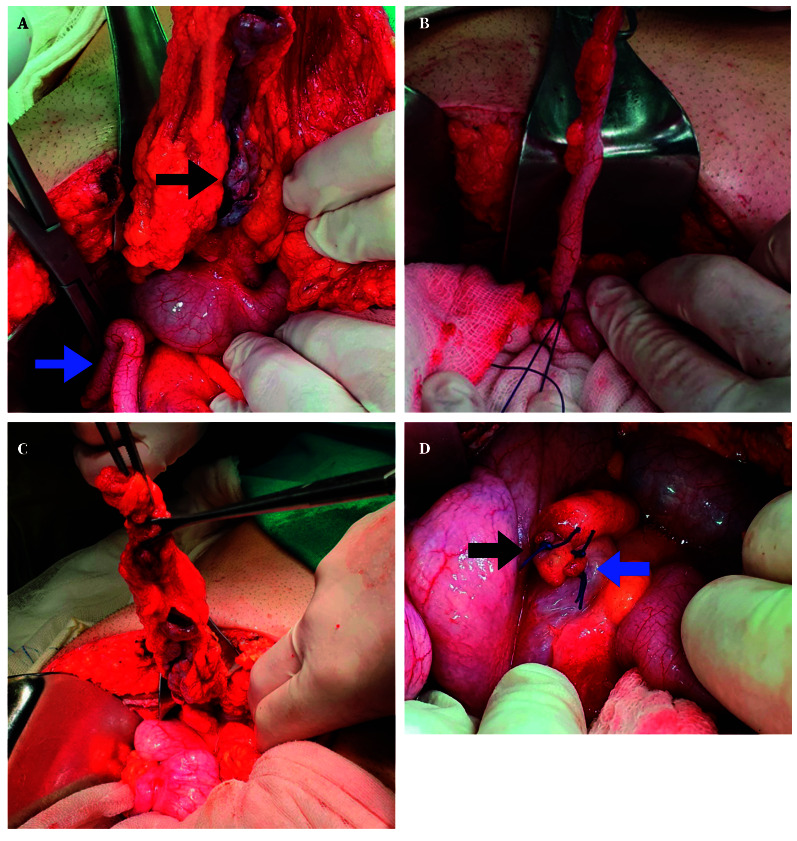
Intraoperative images (laparotomy). **A.** The blue arrow indicates a retrocecal appendage and the black arrow points to a structure presumed to be an anteriorly located appendix. **B.** Dissection of the appendix. **C.** Appendectomies. **D.** Stumps of both tubular structures after ligation.

The patient was admitted with a presumptive diagnosis of acute appendicitis and underwent laparotomy via a median infraumbilical incision. Intraoperatively, serous fluid was found in the right iliac fossa. An 8 cm gangrenous tubular structure covered by momentum was identified anterior to the cecum, and a second 10-cm inflamed tubular structure was observed in the retrocecal region. Macroscopically, the case was classified as a Type 2B appendiceal duplication (Figs. 2A-C). Appendectomy was performed for both appendices, and the proximal stumps were double-ligated (Fig. 2D). A drain was placed in the rectovesical fossa.

Postoperatively, the patient received IV ceftriaxone (1 g twice daily) and metronidazole (500 mg three times daily). Oral feeding was initiated 24 hours after and was well tolerated. As there was no output from the drain by postoperative Day 2, it was removed. The patient experienced no complications and was discharged on postoperative Day 4.

**Figure 3:**
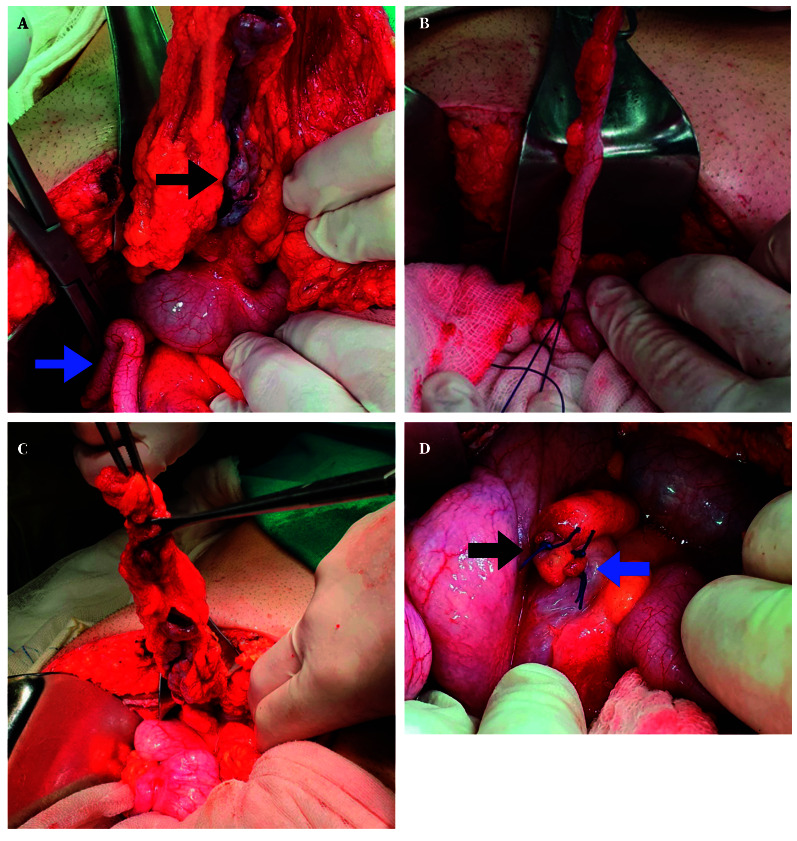
Histopathological light microscopy images. **A.** Vascular structures within a hyalinized stroma (H&E, x20). **B.** Prominent vascular structures within the pseudotumor area (H&E, x20). **C.** Immunoreactivity for CD34 in vascular structures (x20). **D.** Desmin immunostaining in vascular structures (x20).

Histopathological examination of the anteriorly located structure revealed a hyalinized vascular pseudotumor of mesenteric origin, characterized by fibrinopurulent exudate on its external surface and areas of luminal hematoma (Fig. 3). Examination of the retrocecal structure confirmed an inflamed appendix containing a fecalith within its lumen, with hyperemic mucosa and lymphoid hyperplasia.

At the 9-month postoperative follow-up, the patient remained asymptomatic.

## DISCUSSION

Appendiceal duplication is a rare anomaly that may occur at any age and shows a male predominance (male-to-female ratio 1.4:1)^2^. It is typically asymptomatic, with clinical manifestations arising only when acute inflammation develops in one or both appendices. Radiological differentiation of appendiceal duplication from other pathologies can be challenging. CT is considered the most reliable imaging modality, although false-negative results have been reported^3^. In the present case, the young male patient presented with symptoms of acute appendicitis. Targeted CT scan imaging demonstrated two blind-endings, inflamed tubular structures, leading to a preoperative diagnosis of duplicate appendicitis.

In most cases, diagnosis and classification are made intraoperatively. According to the Cave-Wallbridge classification, type B2 duplication is the most commonly reported subtype, in which one appendix occupies its usual position while the other arises along the *taenia* line. In approximately 54% of cases, inflammation is found only in one appendix^2^. Particularly in cases where inflammation affects only the anterior appendix, the other appendix in the retrocecal region may be overlooked. The most frequently performed surgical procedure in the literature is laparotomy. In our case, exploratory laparotomy revealed two inflamed tubular structures - one retrocecal and one anterior to the cecum - consistent with Type B2 duplication.

Condition such as cecal diverticulitis, mesenteric adenitis, and epiploic appendagitis may mimic duplicated appendicitis in the pericecal region, presenting with findings such as omental adhesions, phlegmon, or necrosis. Other differential diagnoses include colon adenocarcinoma, appendiceal tumors, and intussusception, which can form mass-like lesions. When clinical differentiation is uncertain, definitive diagnosis relies on histopathological examination. In this case, the lesion identified as a hyalinized vascular pseudotumor of mesenteric origin uniquely mimicked a second appendix - an association not previously described in the literature. Its cecal proximity, tubular configuration, gangrenous appearance, and associated omental adhesions closely resembled a true appendix, rendering the intraoperative diagnosis particularly challenging. The term *hyalinized vascular pseudotumor* was used by the pathologist to describe a mesenteric lesion with a hyalinized stroma. Although mesenteric inflammatory pseudotumors have been previously reported^8^, this lesion differed histologically due to its dominant vascular component. Thus, the term *hyalinized vascular pseudotumor of mesenteric origin* accurately reflects its distinct pathological features.

This case provides a novel contribution to the differential diagnosis of appendiceal duplication. The coexistence of mesenteric hyalinized vascular pseudotumor and acute appendicitis is exceedingly rare. The case described here - clinically, radiologically, and intraoperatively suggestive of duplicate appendicitis - highlights a diagnostic pitfall for surgeons. Surgeons should maintain a high index of suspicion for such anomalies during appendectomy. Preoperative CT imaging may have limited diagnostic value, and intraoperative macroscopic findings can be misleading. Ultimately, definitive diagnosis requires histopathological confirmation.

## Data Availability

They are available upon request to the corresponding author.
